# Contact inhibition modulates intracellular levels of miR-223 in a p27kip1-dependent manner

**DOI:** 10.18632/oncotarget.1803

**Published:** 2014-03-04

**Authors:** Joshua Armenia, Linda Fabris, Francesca Lovat, Stefania Berton, Ilenia Segatto, Sara D'Andrea, Cristina Ivan, Luciano Cascione, George A. Calin, Carlo M. Croce, Alfonso Colombatti, Andrea Vecchione, Barbara Belletti, Gustavo Baldassarre

**Affiliations:** ^1^ Division of Experimental Oncology 2, CRO, National Cancer Institute, Aviano, Italy; ^2^ Department of Molecular Virology, Immunology and Medical Genetics and Comprehensive Cancer Center, Ohio State University, Columbus, OH, USA; ^3^ Department of Experimental Therapeutics, The University of Texas MD Anderson Cancer Center, TX; ^4^ Department of Biomedical Sciences and Technologies, MATI Center of Excellence, University of Udine, Udine, Italy; ^5^ Division of Pathology, II University of Rome “La Sapienza”, Santo Andrea Hospital, Rome, Italy

**Keywords:** p27kip1, miR-223, RNA binding, contact inhibition

## Abstract

MicroRNAs (miRs) are a large class of small regulatory RNAs that function as nodes of signaling networks. This implicates that miRs expression has to be finely tuned, as observed during cell cycle progression.

Here, using an expression profiling approach, we provide evidence that the CDK inhibitor p27^Kip1^ regulates miRs expression following cell cycle exit. By using wild type and p27KO cells harvested in different phases of the cell cycle we identified several miRs regulated by p27^Kip1^ during the G1 to S phase transition. Among these miRs, we identified miR-223 as a miR specifically upregulated by p27^Kip1^ in G1 arrested cells. Our data demonstrate that p27^Kip1^ regulated the expression of miR-223, via two distinct mechanisms. p27^Kip1^ directly stabilized mature miR-223 expression, acting as a RNA binding protein and it controlled E2F1 expression that, in turn, regulated miR-223 promoter activity. The resulting elevated miR-223 levels ultimately participated to arresting cell cycle progression following contact inhibition. Importantly, this mechanism of growth control was conserved in human cells and deranged in breast cancers.

Here, we identify a novel and conserved function of p27^Kip1^ that, by modulating miR-223 expression, contributes to proper regulation of cell cycle exit following contact inhibition. Thus we propose a new role for miR-223 in the regulation of breast cancer progression.

## INTRODUCTION

MicroRNAs (miRs) are a large class of small regulatory RNAs, ~22 nucleotides long, important for the regulation of numerous biological processes [1]. One of the key and most interesting feature of miRs is their stability. Mature miRs persist for hours, or even days, after their production is arrested [2, 3]. This property has been linked to the necessity of obtaining high miR levels in the cell in order to achieve proper gene regulation [2]. However, many miRs show a dynamic pattern of expression and undergo rapid downregulation during cell cycle progression [4-6]. A large body of literature indicates that miRs control cell cycle progression by direct targeting of critical modulators, such as cyclins, CDKs and CDK inhibitors [7-11]. On the other side, cell cycle progression affects miR stability [4-6] and transcription [8, 11]. Accordingly, cell cycle entry is preceded by induction and/or repression of miRs transcription by selected regulators such a E2F, Myc and p53 [10]. Much less is known about the regulation of miR stability during cell cycle progression and whether this miRs property has a particular significance. It is conceivable that regulatory loops exist to balance the expression of miRs and cell cycle regulators with the ultimate goal of achieving a stringent control of cell proliferation.

Altered cell proliferation is the most obvious hallmark of cancer and cancer cells are characterized by the insensitivity to signals of growth arrest. Nutrient deprivation and contact inhibition are the most common stimuli that arrest normal cell proliferation and that are lost in cancer. In particular, contact inhibition that is the ability of normal cells to restrain continuous growth when neighboring cells touch each other [12], is lost in cancer cells, allowing them to grow in uncontrolled manner even after cell-cell contact is reached. Cell cycle arrest after either serum deprivation [13] or contact inhibition [14] primarily relies on increased expression of the CDK inhibitor p27^kip1^ (hereafter referred to as p27).

The fine tuning of p27 expression during cell cycle has been also ascribed to expression of miRs 221/222 and the significance of this regulation in cancer progression has been well documented [7, 15]. When cells are exposed to mitogenic stimuli p27 protein expression decreases [13, 16] and miR 221/222 expression increases, determining an amplification of the pro-mitogenic stimuli that ultimately favors the entrance in S phase [10, 15]. The regulation of p27 following anti-mitogenic stimuli is far less clear and whether the control of cell cycle exit by p27 is also based on the regulation of miRs expression is not known. Here, we address this particular aspect, studying the fluctuation of miR levels during cell cycle exit, with particular emphasis on the role played by p27.

## RESULTS

### p27 contributes to the regulation of miR-223 expression in G1 arrested cells

**Figure 1:**
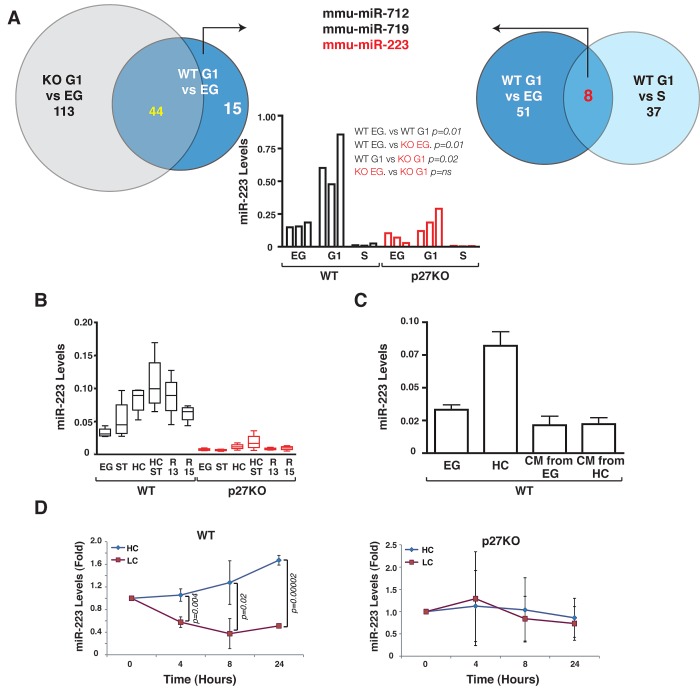
p27 regulates miR-223 expression following contact inhibition (A) Schematic representation of miR expression profile analyses. Venn diagram on the left shows numbers of differentially expressed miR in EG *vs* G1 WT and p27KO MEF. Venn diagram on the right shows numbers of differentially expressed in EG *vs* G1 and S phase *vs* G1 WT MEF. In the graph, qRT-PCR validation of miR-223 expression in the same MEF population used for the array is reported. Data represent the 2^-ΔCT^ values obtained by normalizing miR-223 with snoRNA234 expression. (B) miR-223 expression in 4 independent MEF preparations/genotype, cultured as indicated. Data represent the 2^-ΔCT^ values obtained by normalizing miR-223 with U6 expression. (C) miR-223 expression in 3 independent MEF preparations cultured in EG or HC condition or in EG condition in the presence of conditioned medium (CM) from EG or HC cells as indicated. Data represent the 2^-ΔCT^ values obtained by normalizing miR-223 with U6 expression. (D) miR-223 expression in 3 independent WT (left graph) or p27KO (right graph) MEF preparations cultured in HC, then split and replated at high (HC) or low (LC) confluence for up to 24 hours. Data are expressed as fold increase of miR-223 respect to the T0 (HC harvested cells). *p* values were obtained by unpaired student t-test, using either Excel or Prism softwares. Abbreviations in this figure are: G1= cells arrested in G1 phase of the cell cycle by contact inhibition and serum deprivation for 24 hours; EG = Exponentially growing cells; S= S phase cells collected 10 hours after release from a double thymidine block. ST= Serum Starved; HC= High Confluence; HC ST, grown at high confluence and serum starved; R13/R15= Cells blocked in G1 by serum starvation and high confluence and then released in complete medium for 13 or 15 hours.

In order to identify miRs potentially regulated by p27 in G1 arrested cells we exploited two strategies. First, using primary mouse embryo fibroblasts (MEF) from WT and p27KO embryos we compared the miR expression profiles in cells collected under exponential growth (EG) *vs* G1 arrested (G1). We identified 59 miRs differentially expressed in WT MEFs between EG *vs* G1 (Figure 1A and Supplementary Table 1 and 2). Among these, 15 miRs were not in common with the 157 differentially expressed in EG *vs* G1 p27KO MEFs, thus potentially representing the miRs linked to G1 arrest in a p27-dependent manner (Figure 1A and Supplementary Table 1). Second, we compared miR profiles from WT MEFs in G1 *vs* S phase (S). 45 miRs were differentially expressed (Figure 1A and Supplementary Table 3) and among them, 8 miRs were in common with the 59 identified in WT MEF, EG *vs* G1 group, reasonably representing miRs specifically modulated by G1 arrest. To select only p27-dependent miRs necessary for the G1 arrest, we compared the group of 15 miRs with the group of 8 miRs (Figure 1A and Supplementary Table 4). Three miRs, mmu-miR-223, mmu-miR-712 and mmu-miR-719, were regulated by both G1 arrest and the presence of p27 (Figure 1A and Supplementary Table 4). Among them, mmu-miR-223 (hereafter miR-223) was the only one with an identified human homolog and was therefore chosen for further analyses.

Quantitative RT-PCR (qRT-PCR) analyses performed on RNA from the same MEF preparations (Figure 1A, middle graph) and on 4 other independent MEF preparations/genotype (Figure 1B) confirmed our array data. G1 arrest, induced either by serum deprivation or by contact inhibition, elicited a marked increase of miR-223 levels in WT MEFs (Figure 1B), although only contact inhibition caused statistically significant differences (Figure 1B and Supplementary Table 5). The combined use of serum deprivation and contact inhibition further increased the levels of miR-223 in WT cells (Figure 1B and Supplementary Table 5). Transition from G1 to S phase led to progressive decrease of miR-223 levels, similarly to what observed in EG cells (Figure 1B and Supplementary Table 5). miR-223 levels paralleled the expression of p27 protein, as demonstrated by immunofluorescence (Supplementary Figure 1) or western blot analyses (Figure 3D). When p27KO MEFs were analyzed under the same culture conditions no significant fluctuation in miR-223 levels was observed. Only when contact inhibition and serum deprivation were used together a modest increase in miR-223 expression was appreciated, although it did not reach statistical significance (Figure 1B and Supplementary Table 5).

### p27 is a critical mediator of miR-223 expression after contact inhibition

**Figure 2:**
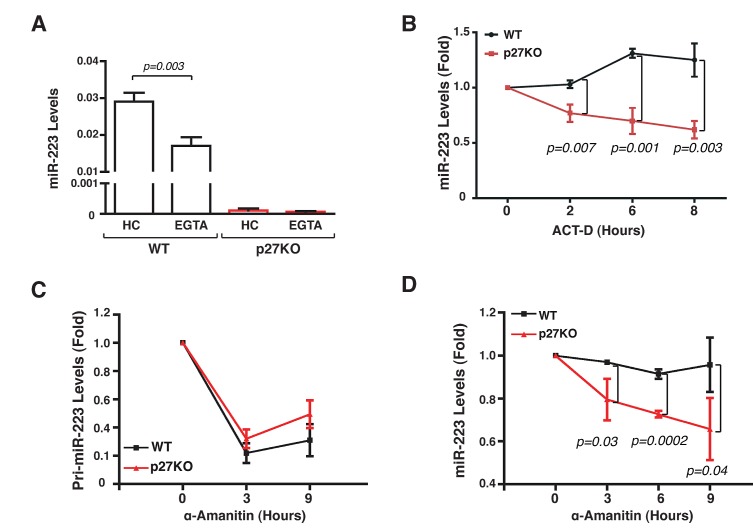
Contact inhibition regulates miR-223 transcription (A) miR-223 expression in 3 independent WT or p27KO MEF preparations cultured in HC condition and then treated with EGTA for 1 hour. Data represent the 2^-ΔCT^ values obtained by normalizing miR-223 with U6 expression. (B) miR-223 expression in 3 independent WT or p27KO MEFs preparation cultured in HC condition in the presence or not of actinomycin-D (ACT-D) for up to 8 hours. Data are expressed as fold increase of miR-223 levels respect to the T0 (HC harvested cells). (C-D) pri-miR-223 (C) or miR-223 (D) expression in 3 independent WT or p27KO MEF preparations cultured in HC in presence or not of α-amanitin, for up to 9 hours. Data are expressed as fold increase of pri-miR-223/miR-223 levels respect to the T0 (HC harvested cells).

Next, we investigated in more detail the regulation of miR-223 by p27 in G1 arrested cells following contact inhibition. By exposing WT MEFs to conditioned medium harvested from WT MEFs under EG or highly confluent (HC) conditions, we excluded that secreted/diffusible factors produced in HC could induce miR-223 expression (Figure 1C). Conversely, by splitting cells from HC culture into low or high confluence conditions (Figure 1D) or by treating HC cells with EGTA to disrupt the cell-cell contacts (Figure 2A), we observed that cell-cell contact was necessary in WT, but not in p27KO, MEFs to sustain the expression of miR-223.

**Figure 3:**
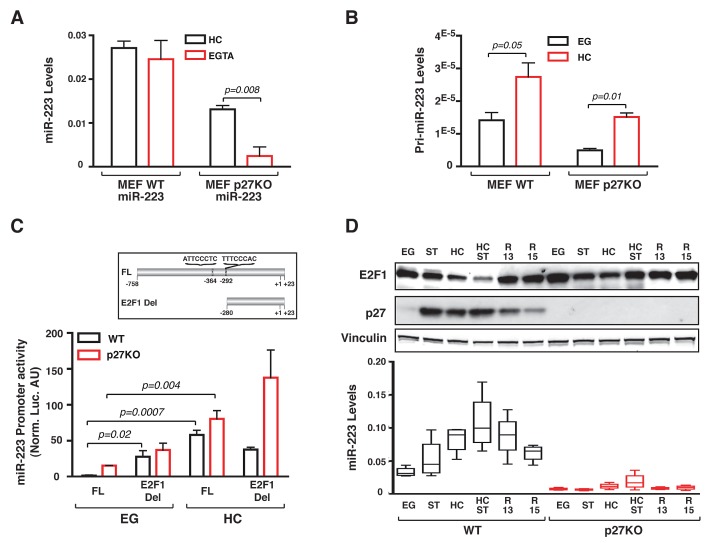
Contact inhibition stimulates miR-223 promoter activity by decreasing E2F1 expression (A) miR-223 expression in WT and p27KO MEFs transduced with lentiviruses encoding for pre-miR-223 under the control of a CMV promoter, cultured in HC condition and then treated with EGTA for 1 hour. Data represent the 2^-ΔCT^ values obtained by normalizing miR-223 with U6 expression. (B) pri-miR-223 normalized expression in 3 independent WT or p27KO MEF preparations, cultured in Exponential Growth (EG) or High Confluence (HC). (C) miR-223 promoter activity in WT and p27KO cells grown in EG or HC conditions. In the inset, miR-223 promoter constructs used are depicted. FL= full length; E2F1 Del= deleted of the 2 putative E2F1 binding sites. (D) Western blot analysis of E2F1 and p27 expression in WT and p27KO MEFs harvested at the indicated culture conditions. Vinculin was used as loading control. In the lower graph, miR-223 expression levels in the same culture conditions are reported. EG = Exponentially growing cells; ST= Serum Starved; HC= High Confluence; HC=ST, starved and grown at high confluence; R13/R15= Cells blocked in G1 by serum starvation and high confluence and then released in complete medium for 13 or 15 hours.

### miR-223 stability is affected by transcriptional and post-transcriptional mechanisms

To dissect the mechanism whereby p27 regulated miR-223 expression following contact inhibition we blocked RNA transcription with Actinomycin D (Act-D) and measured the levels of miR-223. In HC WT MEFs miR-223 remained stable over time, even in the presence of Act-D, while its levels significantly decreased in p27KO cells (Figure 2B). The use of α-amanitin, a specific inhibitor of RNA Polymerase II, which is responsible of miR transcription [17], confirmed these data (Figure 2C and D). α-amanitin completely blocked pri-miR-223 expression both in WT and p27KO MEFs (Figure 2C), but only in p27KO MEFs mature miR-223 levels dropped after treatment (Figure 2D), suggesting that the expression of p27 was necessary for the stability of mature miR-223. Accordingly, when pre-miR-223 was ectopically expressed under the control of an exogenous CMV promoter and cell-cell contacts were disrupted by EGTA treatment, levels of miR-223 remained stable in WT but not in p27KO MEFs (Figure 3A).

### A miR-223/E2F1 regulation loop controls cell cycle exit after contact inhibition

The above data suggested that cell-cell contact triggered miR-223 transcription, which was eventually stabilized by p27 expression. To validate this hypothesis we measured the levels of endogenous pri-miR-223 demonstrating that pri-miR-223 levels increased in HC conditions, in both WT and p27KO cells (Figure 3B). These data implied that in primary MEFs contact inhibition directly signaled on the miR-223 promoter, in a manner at least partially independent from p27 expression. Indeed, achievement of cell confluence increased mouse miR-223 promoter activity in both WT and p27KO cells, as judged by luciferase reporter assay (Figure 3C). It has been proved that the transcription factor E2F1 binds and represses miR-223 promoter in human acute myeloid leukemia (AML) [18] and that miR-223 promoter regulation is conserved among human and mouse [19, 20]. Given the established role of E2F1 in cell cycle progression [21], we tested whether cell-cell contact regulated miR-223 promoter activity via E2F1. Our results indicated that E2F1 expression was regulated by cell-cell contact in WT cells and its levels inversely correlated with those of miR-223 (Figure 3D). The deletion of two putative E2F1 binding sites in the miR-223 promoter (E2F1Del in Figure 3C) significantly increased its activity in WT cells, only when cultured in EG condition. This effect was specifically due to the two predicted-E2F1 binding sites since similar results were obtained when these two E2F1 binding sites were point mutated (Supplementary Figure 2A). In contrast, deletion of E2F1 sites mildly increased miR-223 promoter activity in p27KO cells both in EG and HC, although differences did not reach statistical significance (Figure 3C). This finding was in accord with the level of endogenous protein, showing that in HC p27KO cells E2F1 expression was not completely down modulated (Figure 3D).

Bioinformatic analyses highlighted the presence of a conserved site for miR-223 binding in the mouse E2F1 3'UTR (not shown), as previously demonstrated in human AML [18], supporting the possibility that also in mice a feedback regulation loop may exist. Western blot and qRT-PCR analyses proved that in HC WT MEFs miR-223 knock-down resulted in upregulation of E2F1 (2.9 folds) (Supplementary Figure 2B, left and middle panels). Conversely, in S phase WT MEFs, when miR-223 levels dropped and E2F1 expression raised (Figure 3D), miR-223 overexpression resulted in a significant downregulation of E2F1 protein (Supplementary Figure 2B, right panels).

**Figure 4:**
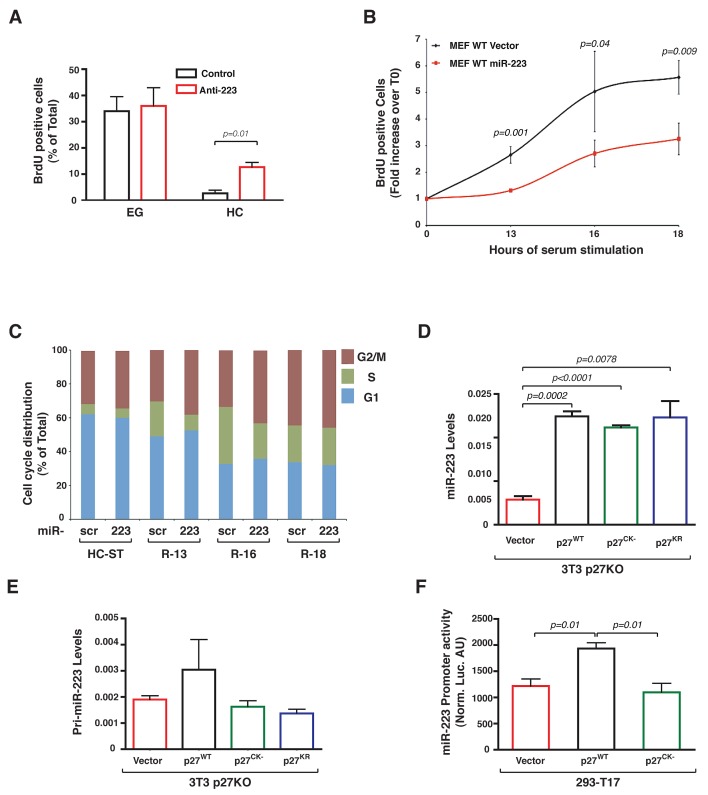
miR-223 controls cell cycle exit in WT MEFs (A) BrdU incorporation assay of WT MEFs transfected with anti-miR-223 (Anti-223) or control oligo (Control), grown at EG or HC. Percentage of BrdU positive cells in each condition from two different MEF preparations is reported. (B) BrdU incorporation assay of WT MEF overexpressing or not miR-223, grown at high confluence, serum starved (HC-ST) and released in complete medium for 13, 16, 18 hours. Data are expressed as fold induction respect to T0 (HC-ST) and represent the mean (±SD) values obtained from three different MEFs preparation. (C) Cell cycle distribution of WT MEF overexpressing or not miR-223, grown at high confluence and serum starved (HC-ST) and released in complete medium for 13, 16, 18 hours (R-13, R-16, R-18), measured by FACS analysis. Data represent the mean values obtained from two different MEFs preparation/treatment. D/E, miR-223 (D) and pri-miR-223 (E) expression in p27KO 3T3 fibroblasts transduced with retroviruses encoding for p27^WT^, p27^CK-^ and p27^KR^, as indicated. Data represent the 2^-ΔCT^ values obtained by normalizing miR-223 with U6 expression (D) or pri-miR-223 expression normalized by the expression of the housekeeping gene GusB (E). (F) miR-223 promoter activity in 293-T17 cells transfected with a control vector or p27^WT^ or p27^CK-^, as indicated. *p* values were obtained by unpaired student t-test, using either Excel or Prism softwares.

Importantly, this feedback regulation loop played a critical role in the regulation of cell cycle entry and exit. In WT cells, miR-223 knock-down partially rescued the G1 arrest imposed by contact inhibition (Figure 4A) and miR-223 overexpression delayed of about 3 hours the S phase entrance of serum starved MEF following serum stimulation, as demonstrated by BrdU incorporation assay (Figure 4B) and FACS analyses of cell cycle distribution (Figure 4C). Our (not shown) and literature data [22] demonstrated that WT and p27KO MEFs equally arrested following contact inhibition, although p27KO cells showed a faster entrance into the cell cycle following serum stimulation [23] and (Supplementary Figure 2C). However, overexpression of miR-223 had only a partial effect on p27KO MEFs cell cycle entry (Supplementary Figure 2C), suggesting that other mechanisms were also implicated or that compensatory mechanisms existed in p27 null cells.

### p27 is a RNA binding protein and directly stabilizes miR-223

Data collected so far could not fully explain the differences observed in the stability of the mature miR-223 between WT and p27KO cells. To get more insights in the regulation of miR-223 stability by p27, we overexpressed p27 wild type (p27^WT^) or p27^CK-^ and p27^KR^ mutants in p27KO 3T3 fibroblasts (Supplementary Figure 3A and B). p27^CK-^ is unable to bind the Cyclin/CDK complexes, thus it does not block the phosphorylation of retinoblastoma (RB) proteins [24, 25]. p27^KR^ carries two point mutations (K165A and R166A) in the nuclear localization signal and therefore preferentially locates into the cytoplasm [26]. All exogenously expressed p27 proteins were able to significantly increase the levels of endogenous miR-223 in p27KO cells (Figure 4D), demonstrating that the extent of miR-223 increase was not influenced by the loss of nuclear localization or by the loss of cyclin/CDK binding ability and suggesting that p27 stabilized miR-223 expression via a cytoplasmic and CDK-independent activity. Next, we tested the levels of pri-miR-223 in the same cells to verify if p27 could act as a transcriptional regulator, independently on its cyclin/CDK binding, as recently suggested [27]. Our results indicated that only cells expressing the p27^WT^ protein displayed higher levels of pri-miR-223 (Figure 4E). Accordingly, when p27^WT^ or p27^CK-^ were transfected with the miR-223 promoter in 293-T17 cells we observed that only the WT protein was able to significantly increase its transcriptional activity (Figure 4F). Altogether, these data suggested that p27 contributes to regulation of miR-223 transcription via a CDK-dependent nuclear activity, likely via E2F1 inhibition and that p27 could also regulate mature miR-223 expression with a CDK-independent cytoplasmic activity.

**Figure 5:**
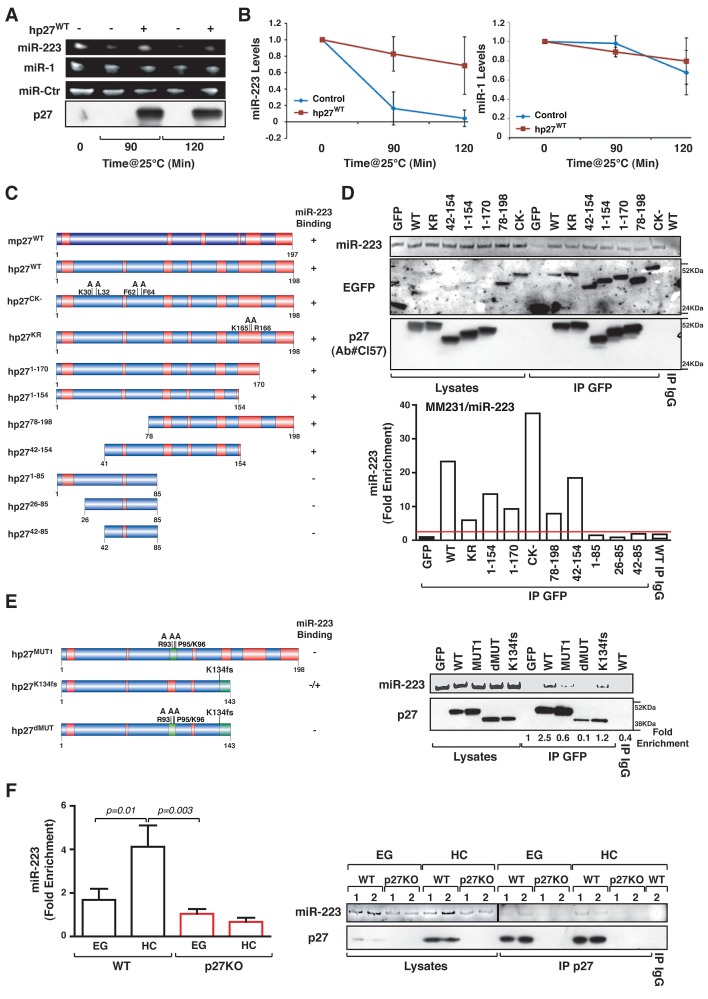
p27 binds and stabilizes mature miR-223 (A) *In vitro* miR degradation assay. Expression of miR-223, miR-1, control miR and p27 protein after 0, 90 and 120 minutes of incubation with lysate from p27KO cells is reported. Human recombinant p27 protein was added where indicated. (B) Quantification of miR-223 (left graph) and miR-1 (right graph) levels from the *in vitro* miR degradation assay described in (A). Data represent the mean of three experiments performed in duplicate and are expressed as fold of miR levels respect to T0. (C) Schematic representation of EGFP-fusion proteins used in RIP assays. (D) Expression of miR-223 and p27 protein using the anti-EGFP or the anti-p27 antibodies (Ab#Cl57), in lysates and RIP from MDA-MB-231-miR-223 cells transfected with the indicated p27 constructs and immunoprecipitated using an anti-EGFP or control IgG antibodies, as indicated. In the lower graph qRT-PCR analyses, evaluating miR-223 binding to p27^WT^ or mutant proteins expressed as fold enrichment respect to miR-223 binding to EGFP transfected cells. (E) Expression of miR-223 and p27 protein using the anti-p27 antibodies (Ab#Cl57), in lysates and RIP from MDA-MB-231-miR-223 cells transfected with the indicated p27 constructs and immunoprecipitated using an anti-EGFP or control IgG antibodies, as indicated. The fold enrichment value (calculated as in D) is reported under the blot. (F) qRT-PCR analyses evaluating miR-223 binding to endogenous p27 protein expressed as fold enrichment respect to miR-223 binding to control IgG in WT and p27KO cells, cultured in exponential growth (EG) or at high confluence (HC). On the right, expression of miR-223 (upper panel) and p27 protein (lower panel) is reported. Different MEF preparations (1 and 2) are indicated. *p* values were obtained by unpaired student t-test, using either Excel or Prism software.

These data prompted us to directly test whether p27 affected miR-223 stability. To this aim, we used an *in vitro* degradation assay in which cell lysates from p27 null cells were used to test the half-life of a synthetic miR-223 oligo, in the presence or not of recombinant p27. This assay demonstrated that recombinant p27 protein was able to significantly lengthen the half-life of miR-223, but not of control-miR or of the un-related miR-1 (Figure 5A and B), suggesting that p27 directly bound and protected from degradation miR-223. This possibility was supported by *in silico* analyses (BindN prediction program), showing that both human and mouse p27 proteins contains several putative RNA-binding domains (Figure 5C and Supplementary Figure 3). Using RNA-immunoprecipitation (RIP) assay, we could assess that human p27 was able to bind miR-223 in a cytoplasmic and CDK-independent manner, by a region comprised between aminoacids (aa) 86 and 154 (Figure 5D and Supplementary Figure 3C-E). Two putative RNA-binding sites are present in this region and one of them is highly conserved among mammals (Supplementary Figure 3F). The most conserved RNA-binding site was the region comprised between aa 90 and 103 (Figure 5D and Supplementary Figure 3F). *In silico* analyses demonstrated that three point mutations in R93, P95 and K96 would be sufficient to completely disrupt this binding site (Supplementary Figure 3G). We thus generated this mutant (hereafter called p27^MUT1^) and demonstrated that it barely binds miR-223, with an affinity below the threshold we set as significant when the fold enrichment was calculated (Figure 5E). Next, we tested the possibility that also the region between aa 134 and 142 could bind miR-223. In doing so, we took advantage of the notion that in human breast cancer it has been recently isolated a mutant form of p27 carrying one base deletion at codon 134, resulting in the frameshift of p27 open reading frame and in a truncated protein [28]. We generated this mutant, named p27^K134fs^ and our RIP assay demonstrated that it is still able to bind miR-223, although with very low affinity (Figure 5E). When the two RNA-binding site where concomitantly destroyed (p27^dMUT^ mutant) (Figure 5E and Supplementary Figure 3F) we observed no binding between p27 and miR-223. These data suggested that miR-223 could primarily bind the region between aa 90 and 103 that it is highly conserved among species, and that the region between aa 134 and 142 could participate in the binding (Supplementary Figure 3F). Then, a RIP performed on endogenous p27 immunoprecipitated from HC or EG WT and p27KO MEFs proved the physiological significance of this interaction showing that miR-223 was readily recovered only in WT HC cells (Figure 5F).

### miR-223 reduces cell proliferation in K-RasV12 transformed cells

To understand if the regulation of miR-223 by p27 played a role during transformation, we looked at miR-223 expression in p27WT and p27KO 3T3 fibroblasts transformed with the oncogenic form of K-Ras4B (K-Ras^V12^) (Supplementary Figure 4A), since p27 expression is maintained and has a particular relevance in K-Ras^V12^ transformed cells [29]. Interestingly, p27WT K-Ras^V12^ displayed 3- to 4-folds higher levels of miR-223 compared to p27KO K-Ras^V12^ cells (Supplementary Figure 4B) and a drop of S phase cells from 12% to 7%, following contact inhibition (Supplementary Figure 4D). Conversely, p27KO K-Ras^V12^ cells had a higher percentage of S phase population respect to p27WT K-Ras^V12^ cells, in exponentially growing condition (25% *versus* 12%) and continued to proliferate despite contact inhibition, even if at a reduced extent (Supplementary Figure 4D). The overexpression of miR-223 in p27KO growing cells (Supplementary Figure 4C) strongly reduced the S phase population in p27KO K-Ras^V12^ cells exponentially growing (from 25% to 16%), therefore mimicking the achievement of contact inhibition (Supplementary Figure 4D), while it had only minor effects on cells after achievement of confluence (Supplementary Figure 4D). No significant difference in cell cycle distribution was observed in WT cells when miR-223 was overexpressed (not shown). These data suggested that in the context of cell transformation the regulation of miR-223 expression by p27 played a functional role.

### Regulation of miR-223 by p27 in breast cancer

**Figure 6:**
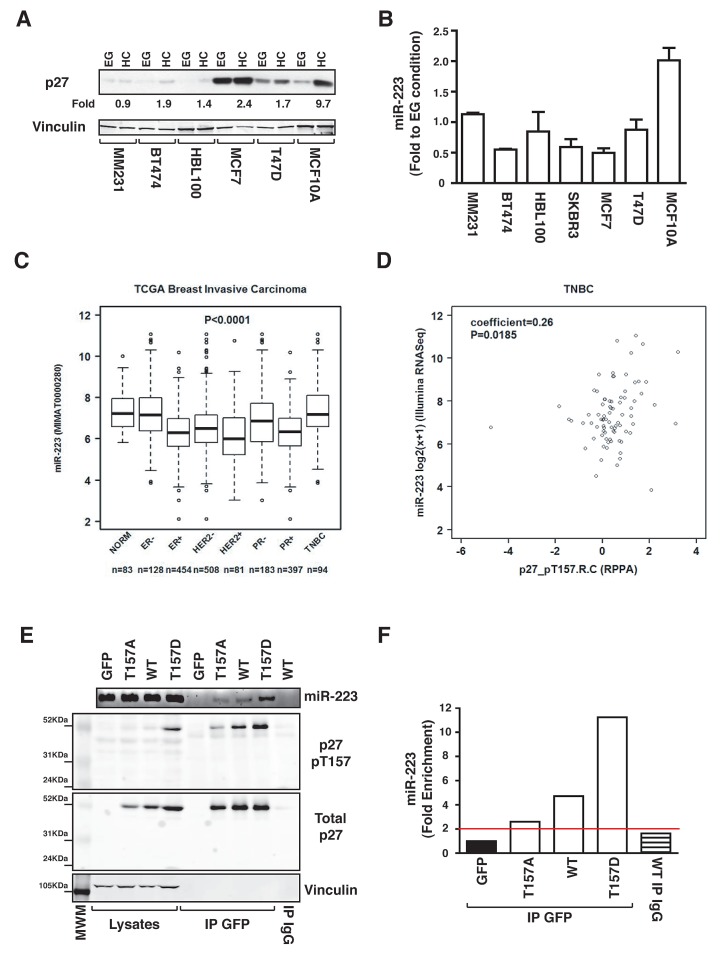
p27-miR-223 axis is deregulated in breast cancer (A) Western blot analysis of p27 expression in breast cancer cell lines, harvested in exponential growth (EG) or at high confluence (HC). Increase of p27 expression in cells grown at HC, expressed as fold increase respect to EG condition, is reported in the figure. Vinculin was used as loading control. (B) miR-223 expression in the indicated breast cancer cell lines grown at HC. Data are expressed as fold increase of miR-223 levels in HC respect to EG. (C) miR-223 expression in the TCGA breast cancer dataset. (D) Correlation between miR-223 and p27-pT157 expression in the TCGA breast cancer dataset, using the Spearman's correlation test. (E) Expression of miR-223, p27 T157 and p27 in lysates and RIP from MDA-MB-231-miR-223 cells, transfected with the indicated EGFP-p27 constructs and immunoprecipitated using an anti-EGFP or control IgG antibodies, as indicated. (F) miR-223 expression in RIP described in (E), evaluating binding to p27^WT^ or mutant proteins and expressed as fold enrichment respect to miR-223 binding in EGFP transfected cells.

Loss of contact inhibition is a hallmark of cancer and the role of p27 is of particular importance during breast cancer progression. The CDKN1B gene, encoding for p27, has been found mutated in some histotypes and deregulated in others [30, 31]. Moreover, p27 deregulated expression is often considered a negative and independent prognostic factor in cancer. Thus, we evaluated the expression of p27 and miR-223 in a panel of breast cancer derived cell lines, grown in EG or HC. p27 was considerably upregulated by contact inhibition only in fibroadenoma-derived MCF10A cells (a model of semi-normal mammary gland epithelial cells) (Figure 6A) and this was paralleled by upregulation of miR-223. In contrast, in tumor-derived cells, miR-223 expression was not modified by contact inhibition (Figure 6B), suggesting that p27 could affect miR-223 regulation in mammary epithelial cells and that it could contribute to loss of miR-223 in breast cancer. To test this hypothesis, we interrogated the Cancer Genome Atlas data set (TCGA), containing miR expression in 83 normal breast tissues and 697 breast cancers[32]. miR-223 levels were generally reduced in the different types of breast cancers, with the exception of TNBC (Figure 6C and Supplementary Figure 5A). Next, we looked at the expression and phosphorylation of p27 in the same data set (403 cancer specimens analyzed by reverse-phase protein array, RPPA) [32]. Our analyses highlighted that, although p27 levels were higher in ER+ and PR+ breast cancers (Supplementary Figure 5B), its phosphorylation on T157 (Supplementary Figure 5C) and T198 (Supplementary Figure 5D) was higher in TNBC. Interestingly, phosphorylation on T157, but not on T198, significantly correlated with miR-223 expression in TNBC (Figure 6D). T198 phosphorylation has been linked to p27 degradation [24] while T157 phosphorylation has been linked to p27 displacement in the cytoplasm [33], suggesting that the latter could participate in the regulation of miR-223 binding. To evaluate this hypothesis, we generated a non-phosphorylable T157 mutant (p27^T157A^) and a pseudo phosphorylated mutant (p27^T157D^) to be tested in RIP assays (Figure 6E). Our results showed that p27 ability to bind miR-223 paralleled the levels of T157 phosphorylation, with the p27^T157D^ mutant displaying the greatest ability (Figure 6E and F), thus providing an explanation for the positive correlation observed in breast cancers (Figure 6D).

## DISCUSSION

Here, we show that p27 controls cell cycle exit following contact inhibition also by a direct regulation of miRs expression. Among the different p27-regulated miRs during G1 arrest, we focused our studies on the regulation of miR-223 and demonstrated that sustained expression of miR-223 represents an important event in the regulation of contact inhibition, both in normal and in K-Ras transformed cells.

Our data convincingly establish that p27 regulates miR-223 expression in two ways. The first is the direct binding of p27 to mature miR-223 that protected miR-223 from degradation. We confirmed this mechanism *in vitro* (Figure 5A-B) and *in vivo* (Figure 5C-F) and observed that it is conserved in mouse and human (Figure 5C-F). We identified two possible miR-223 binding motifs (RNA-BM) in human p27: the first (1^st^ RNA-BM) between aa 90-103 and the second (2^nd^ RNA-BM) between aa 134-142. Interestingly, two point mutations within the 1^st^ RNA-BM (P95S and K96Q) have been found in MEN syndromes [34]. The K96Q substitution, predicted to alter the miR-223 binding motif in p27 (BindN analysis) is also associated with the presence of breast cancer in MEN patients [34]. More intriguingly, two frame shifts mutations (K134fs and P137fs) found in luminal A breast cancer [28, 31] completely disrupt the 2^nd^ RNA-BM reinforcing the potential significance of the regulation of miR-223 stability by p27 in breast cancer onset and/or progression.

The second way by which p27 regulates miR-223 is related to the ability to inhibit pRB phosphorylation by CDKs, thus eventually blocking E2F1 activity. We discovered an auto regulatory feedback loop between E2F1 and miR-223, in which E2F1 repressed the miR-223 promoter and miR-223 downregulated E2F1 expression. This mechanism was effective in WT but not in p27KO cells following contact inhibition and it is conserved from mouse to human [18]. Our data, showing that in p27KO cells contact inhibition failed to properly downregulate E2F1, add new elements to the mechanism by which this crucial transcription factor is regulated and open interesting lines of future investigation. The observation that only p27^WT^ but not the p27^CK-^ mutant is able to stimulate the activity of miR-223 promoter and increase the levels of pri-miR-223 (Figure 4) strongly suggests that p27 participates in the control of miR-223 transcription via the inhibition of E2F1 activity through the CDK/RB pathway. Still, it is possible that p27 contributes to the regulation of miR-223 transcription also via E2F1 unrelated mechanisms, as recently demonstrated for other protein coding genes [27].

Our data are in accord with the phenotypes of p27 [22] and miR-223 [35] knock-out animals, both presenting features of hyper proliferation. Future investigation will possibly establish whether the new mechanism highlighted here plays a role in the physiology/pathology of lymphoid organs, particularly enlarged in both p27 and miR-223 knock-out mice [22, 35].

**Figure 7:**
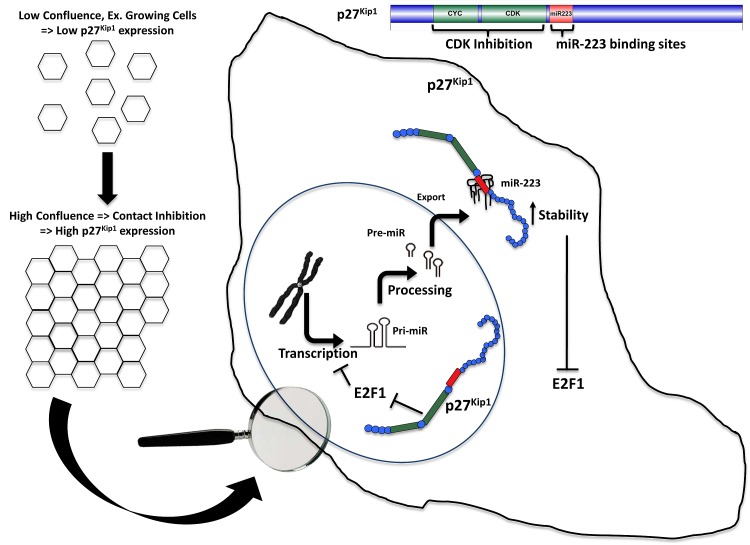
Schematic representation of the proposed mechanism, linking p27 to miR-223 in the control of cell proliferation In normal cells attainment of cell-cell contact determines an increased expression of both cytoplasmic and nuclear p27. Cytoplasmic p27 directly binds and stabilizes miR-223, while nuclear p27, by inhibiting E2F1 expression and activity, induces miR-223 transcription. miR-223 high levels then target E2F1 mRNA, thereby promoting cell cycle arrest. In cancer cells the mechanism is lost, due to the low (or absent) expression levels of p27 and to its T157 phosphorylation levels.

In summary, we show that contact inhibition induces the nuclear and cytoplasmic accumulation of p27, both impinging on miR-223 expression. Nuclear p27 likely counteracts E2F1 activity by impairing RB phosphorylation, thereby increasing miR-223 transcription. Cytoplasmic p27 directly binds mature miR-223 increasing its stability (Figure 7). Future investigation will address if and how post-translational modifications of p27 following contact inhibition can modulate its ability to bind and stabilize miR-223. It is conceivable that one of these modifications might be the phosphorylation of p27 on T157 that correlates with miR-223 levels in TNBC and significantly increases the binding of p27 to miR-223 *in vitro*.

In conclusion, we highlight here a new pathway that participates to the control of cell proliferation both *in vitro* and *in vivo*, representing a promising field of future investigation for cancer research and anti-cancer therapeutics, especially in breast cancer.

## MATERIALS AND METHODS

Detailed description of methods used in this work can be found in the Supplementary Material and Methods section, available online.

### Cell cultures

Primary wild type (WT) and p27 knock-out (p27KO) MEF and 3T3 cells were prepared and cultured as previously described [36]. 293T/17 were used for the production of retroviral particles and cultured in DMEM supplemented with 10% FBS (Sigma). Normal breast and tumor derived cells were cultured as described in SI. All utilized cell lines were authenticated by BMR Genomics srl, Padova, Italia, on January 2012 according to Cell ID™ System (Promega) protocol and using Genemapper ID Ver 3.2.1 to identify DNA STR profiles.

### mmu-miR-expression profile and qRT-PCR

Total RNA (5 μg) was reverse transcribed using biotin end-labeled random octamer oligonucleotide primers. Hybridization of biotin-labeled complementary DNA was performed using a custom miRNA microarray chip (OSU-CCC Human and Mouse MicroRNA Microarray Version 4.0) as described in SI. The miRNA expression data have been submitted to the Gene Expression Omnibus (GEO) with accession number GSE 45538.

### Vectors

Retroviral vectors (MSCV; Clontech) or EGFP-tagged vectors encoding for human p27^WT^ or mutant p27^CK-^ (R30, L32, F62, F64 in Alanine) mutant p27^KR^ (K165, R166 in Alanine) were produced by site directed mutagenesis, as previously described [24].

The EGFP-tagged p27 deletion mutants were previously described [36] or generated by PCR starting from human p27 cDNA. EGFP-tagged p27^CK-^, p27^KR^, p27^MUT1^, p27^K134fs^, and p27d^MUT^ mutants were produced by site directed mutagenesis, as better described in the Supplementary Material and Methods section.

### RNA Immunoprecipitation

RNA immunoprecipitation assay (RIP) was performed essentially as previously reported [37] and better described in SI.

### TCGA statistical analyses

Clinical, miRNA expression and Reverse Phase Protein Array (RPPA) data from TCGA (The Cancer Genome Atlas) available on line at: https://tcga-data.nci.nih.gov/docs/publications/brca_2012/ were downloaded and all statistical analyses were performed in R (ver 2.14.2) (http:///www.r-project.org/).

## SUPPLEMENTARY FIGURES, MATERIALS AND TABLES






