# Supplementary material for: Contact inhibition modulates intracellular levels of miR-223 in a p27kip1-dependent manner
Source: Oncotarget. 2014 Mar 4;5(5):1185–97. doi: 10.18632/oncotarget.1803 (PMC4012735; doi:10.18632/oncotarget.1803)
Supplement: Supplementary file 1 [file oncotarget-05-1185-s001.pdf]

Supplementary Data

Contact inhibition modulates intracellular levels of miR-223 in a p27kip1-dependent manner

Joshua Armenia, Linda Fabris, Francesca Lovat, Stefania Berton, Ilenia Segatto, Sara D'Andrea, Ivan Cristina, Luciano Cascione, George A. Calin, Carlo M. Croce, Alfonso Colombatti, Andrea Vecchione, Barbara Belletti and Gustavo Baldassarre.

Supplementary Figures

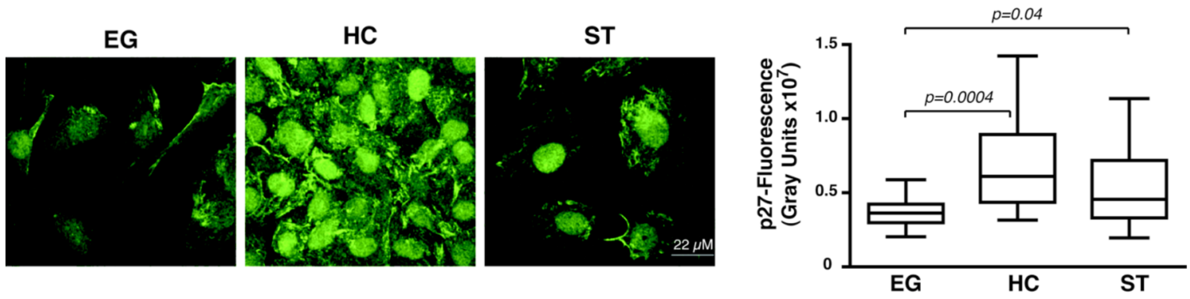

Figure S1. *p27 expression is strongly upregulated in MEFs grown at high confluence.* Immunofluorescence analyses of p27 expression and localization (green) in primary MEFs fixed in exponential growth (EG), high confluence (HC) or serum starved (ST) conditions. On the right, box plot quantification of p27 fluorescence, calculated using the Volocity program (Perkin Elmer) in at least 50 independent cells/condition, expressed as fluorescence gray units. Significance was calculated using the Mann-Whitney unpaired t-test.

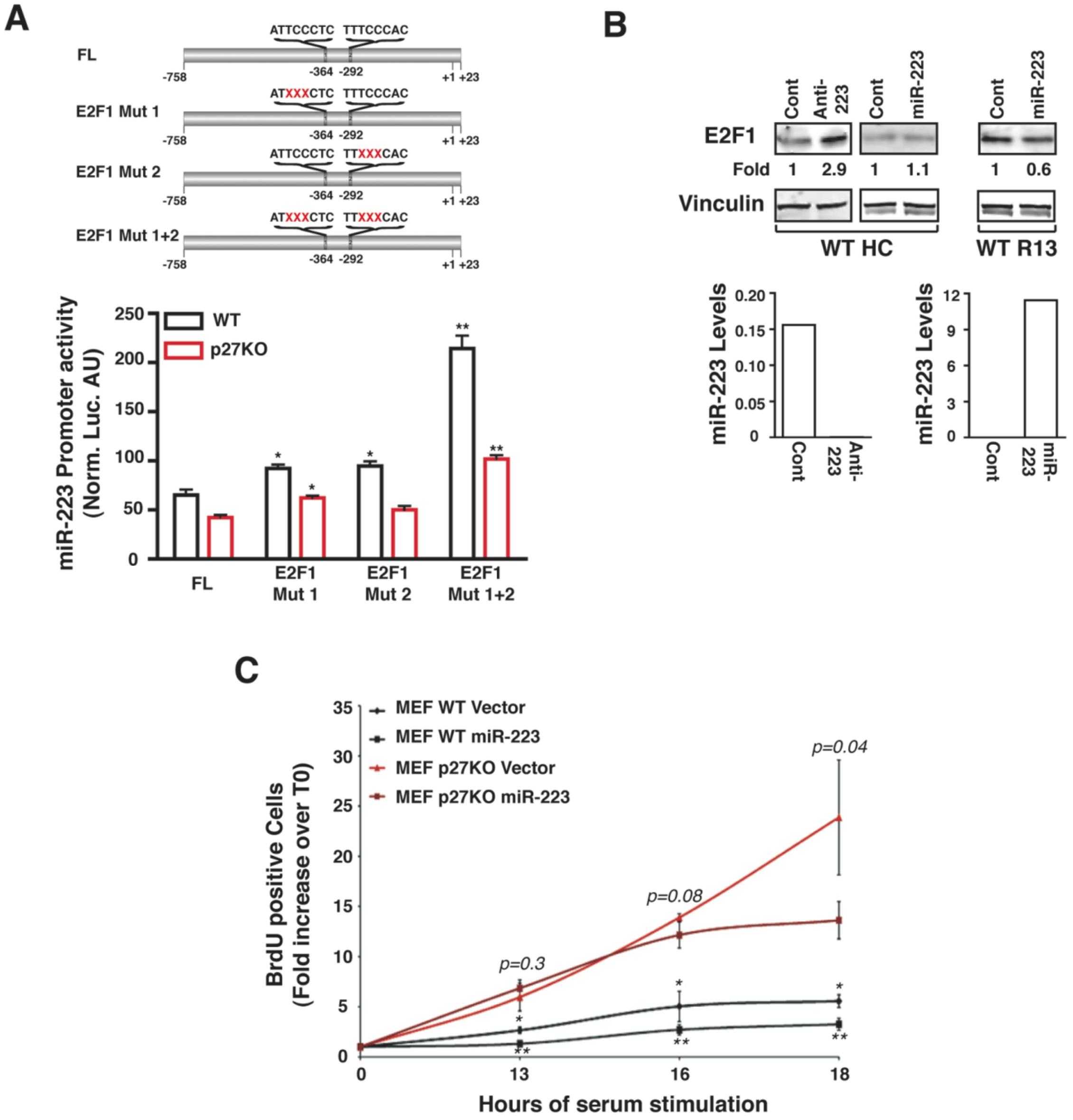

Figure S2. *miR-223 regulates E2F1 expression and cell cycle exit following contact inhibition.* (**A**). miR-223 promoter activity in WT and p27KO cells. On the top, the miR-223 promoter constructs used are depicted. FL= full length; E2F1 Mut 1= mutation of the first E2F1 binding site; E2F1 Mut 2= mutation of the second E2F1 binding site; E2F1 Mut 1+2= mutation of both E2F1 binding sites. **(B).** Western blot analyses of E2F1 expression in WT HC and WT R13 cells transfected with anti-miR or miR mimics, as indicated. In the lower graph, levels of miR-223 in control and transfected cells are reported. Data represent the $2^{-\Delta CT}$ values obtained by normalizing miR-223 with U6 expression. (**C**). BrdU incorporation assay of WT and p27KO MEF overexpressing or not miR-223, grown at high confluence and serum starved (HC-ST) and released in complete medium for 13, 16, 18 hours. Data are expressed as fold induction respect to T0 (HC-ST) and represent the mean (±SD) of three different MEFs preparation.

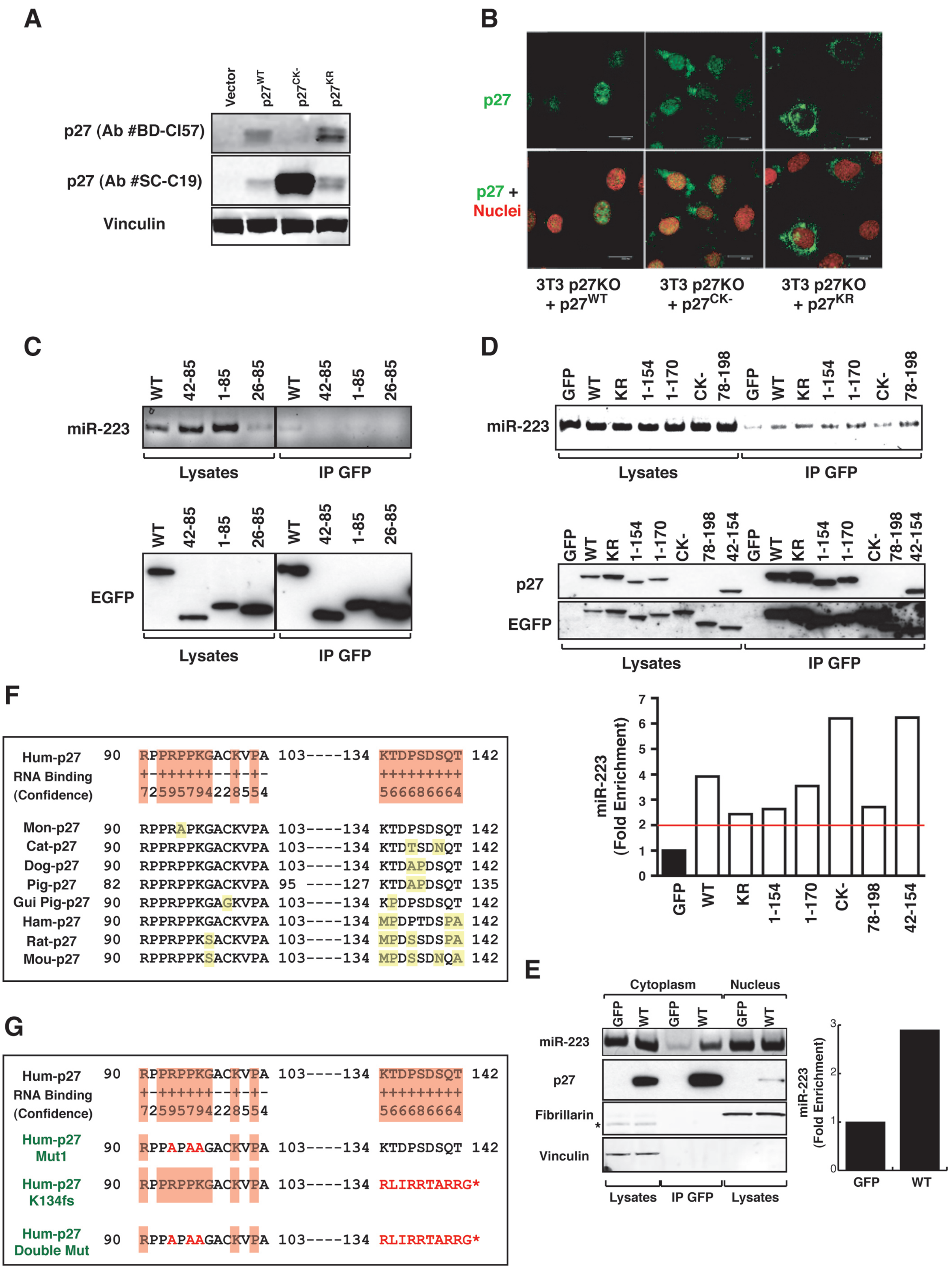
A
Vector
p27WT
p27CK-
p27KR
p27 (Ab #BD-Cl57)
p27 (Ab #SC-C19)
Vinculin
B
p27
p27 + Nuclei
3T3 p27KO + p27WT
3T3 p27KO + p27CK-
3T3 p27KO + p27KR
C
WT 42-85 1-85 26-85 WT 42-85 1-85 26-85
miR-223
Lysates
IP GFP
EGFP
D
GFP WT KR 1-154 1-170 CK- 78-198 GFP WT KR 1-154 1-170 CK- 78-198
miR-223
GFP WT KR 1-154 1-170 CK- 78-198 42-154 GFP WT KR 1-154 1-170 CK- 78-198 42-154
p27
EGFP
miR-223 (Fold Enrichment)
0 1 2 3 4 5 6 7
GFP WT KR 1-154 1-170 CK- 78-198 42-154
F
Hum-p27 90 RPPRPPKGACKVPA 103----134 KTDPSDSQT 142
RNA Binding +-++++++--+-+-
(Confidence) 72595794228554 566686664
Mon-p27 90 RPPRAPKGACKVPA 103----134 KTDPSDSQT 142
Cat-p27 90 RPPRPPKGACKVPA 103----134 KTDTSDNQT 142
Dog-p27 90 RPPRPPKGACKVPA 103----134 KTDAPDSQT 142
Pig-p27 82 RPPRPPKGACKVPA 95 ----127 KTDAPDSQT 135
Gui Pig-p27 90 RPPRPPKGAGKVPA 103----134 KPDPSDSQT 142
Ham-p27 90 RPPRPPKGACKVPA 103----134 MPDPTDSPA 142
Rat-p27 90 RPPRPPKSACKVPA 103----134 MPDSSDSPA 142
Mou-p27 90 RPPRPPKSACKVPA 103----134 MPDSSDNQA 142
E
Cytoplasm
Nucleus
GFP WT GFP WT GFP WT
miR-223
p27
Fibrillarin
*
Vinculin
Lysates
IP GFP
Lysates
miR-223 (Fold Enrichment)
0 1 2 3
GFP WT
G
Hum-p27 90 RPPRPPKGACKVPA 103----134 KTDPSDSQT 142
RNA Binding +-++++++--+-+-
(Confidence) 72595794228554 566686664
Hum-p27 Mut1 90 RPPAPAAGACKVPA 103----134 KTDPSDSQT 142
Hum-p27 K134fs 90 RPPRPPKGACKVPA 103----134 RLIRRTARRG*
Hum-p27 Double Mut 90 RPPAPAAGACKVPA 103----134 RLIRRTARRG*

Figure S3. *p27 binds to miR-223 in a conserved region between aminoacids 90-102.* (**A**). Western blot analyses of p27 expression in 3T3 p27KO fibroblasts transfected with the indicated p27 mutants. Two different anti-p27 antibodies were used to recognize mutants: Ab#BD-Cl57, raised against the CDK binding domain of p27, does not recognize the CK- mutant. Ab#SC-C19, raised against the C-terminus of p27, recognizes the CK- mutant. (**B**). Immunofluorescence analyses of p27 expression and localization (green) in 3T3 p27KO, transfected as indicated. The propidium iodide staining of the nuclei is shown in red. (**C**). Expression of miR-223 and p27 protein using the anti-EGFP antibody in lysates and IP from MDA-MB-231 cells transfected with miR-223 and the indicated p27 constructs. (**D**). Expression of miR-223 and p27 protein using the anti-EGFP antibody in lysates and IP from HT-1080 cells transfected with miR-223 and the indicated p27 constructs. Graph at bottom reports the qRT-PCR analyses evaluating miR-223 binding to $p27^{WT}$ or mutant proteins, expressed as fold enrichment respect to miR-223 binding in GFP transfected cells. (**E**). Expression of miR-223 and p27 protein using the anti-p27 antibody in nuclear or cytosol fractions and IP of the cytosol fraction from MDA-MB-231 cells transfected with miR-223 and GFP or GFP-$p27^{WT}$ constructs. Graph on the right reports the qRT-PCR analyses evaluating miR-223 binding to $p27^{WT}$ or mutant proteins, expressed as fold enrichment respect to miR-223 binding in GFP transfected cells. Fibrillarin and Vinculin expression was used to determine the purity of nuclear and cytosol fraction, respectively. * marks non specific bands recognized by the anti-Fibrillarin antibody in the cytosol fraction. (**F**). Comparison of p27 protein sequence among mammals in the two putative miR-223 binding domains. The aminoacids predicted to bind RNA in human p27 using the BindN resource are highlighted in red (score 1 to 9, with 1=lowest and 9=highest value). Aminoacid substitutions in the different mammals are highlighted in yellow. (**G**). Schematic representation of the 3 mutants used to precisely map the miR-223 binding domain in p27. The $p27^{MUT1}$ construct carries three substitutions (R93A, P95A and K95A) that completely alter the RNA binding domain between aa 90 and 103 as predicted using the BindN resource. The $p27^{K134fs}$ construct carries one base deletion (A420) resulting in the frame shift of the protein from K134 and in the generation of a truncated protein of 143 aa that lost the RNA binding domain between aa 134 and 143 (the alternate frame is reported in red). The $p27^{dMUT}$ (double mutant) construct carries both RNA binding domains deletions.

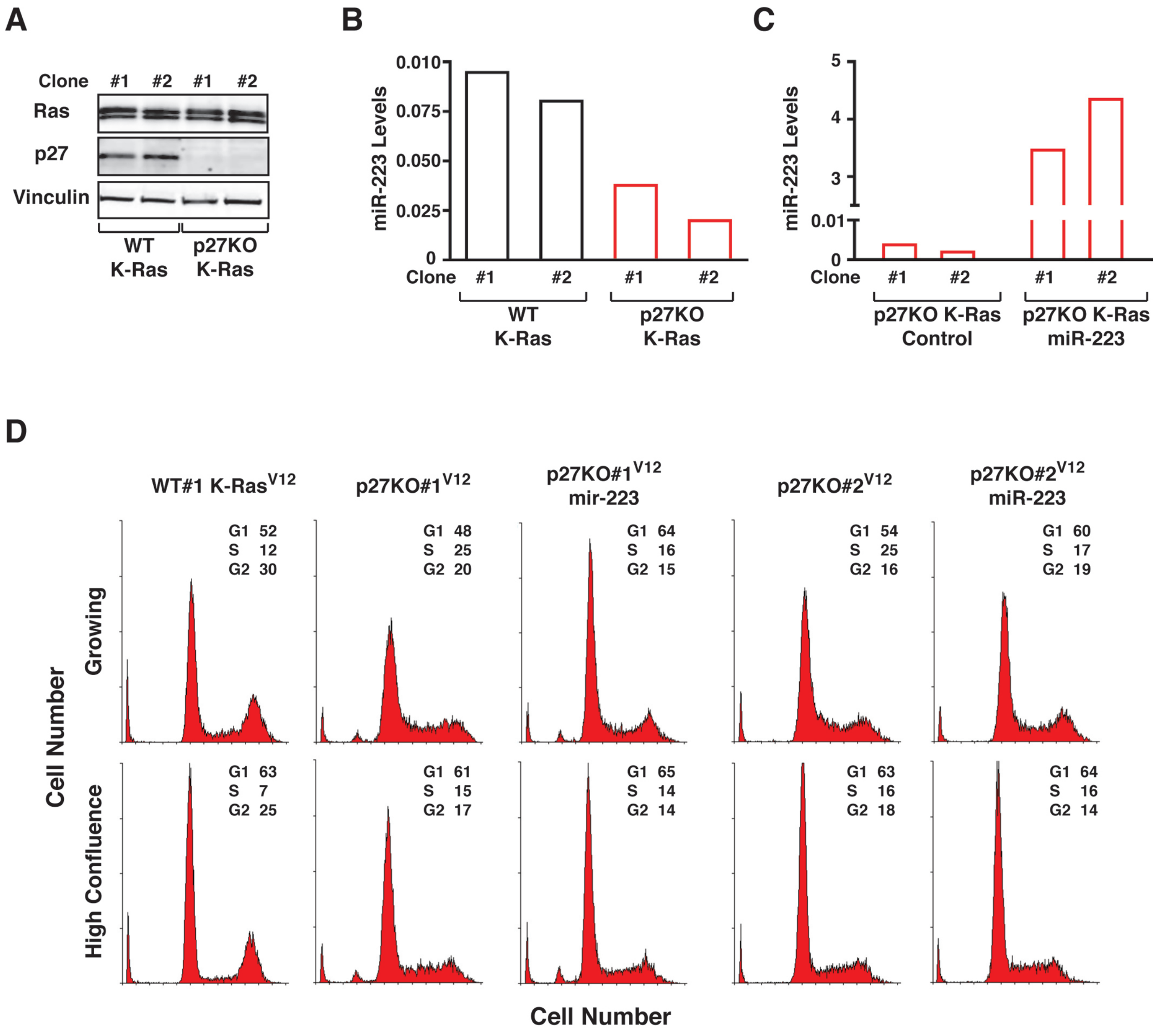

Figure S4. *miR-223 expression reduce the hyper proliferation of K-Ras transformed p27KO cells.* (**A**). Western blot analyses of Ras and p27 expression in 2 different pools of 3T3 WT and p27KO fibroblasts transformed with the K-Ras^{V12} oncogene. Vinculin was used as loading control. (**B**). Normalized miR-223 expression in the K-Ras^{V12} transformed cell clones described in (A). (**C**). Normalized miR-223 expression in the p27KO K-Ras^{V12} transformed cell clones transfected with control miR or miR-223 as indicated. (**D**). FACS analysis of DNA content in WT and p27KO K-Ras^{V12} transformed fibroblasts in exponentially growing conditions (Growing) or after the achievement of confluence (High confluence) and transfected with miR-223 or control.

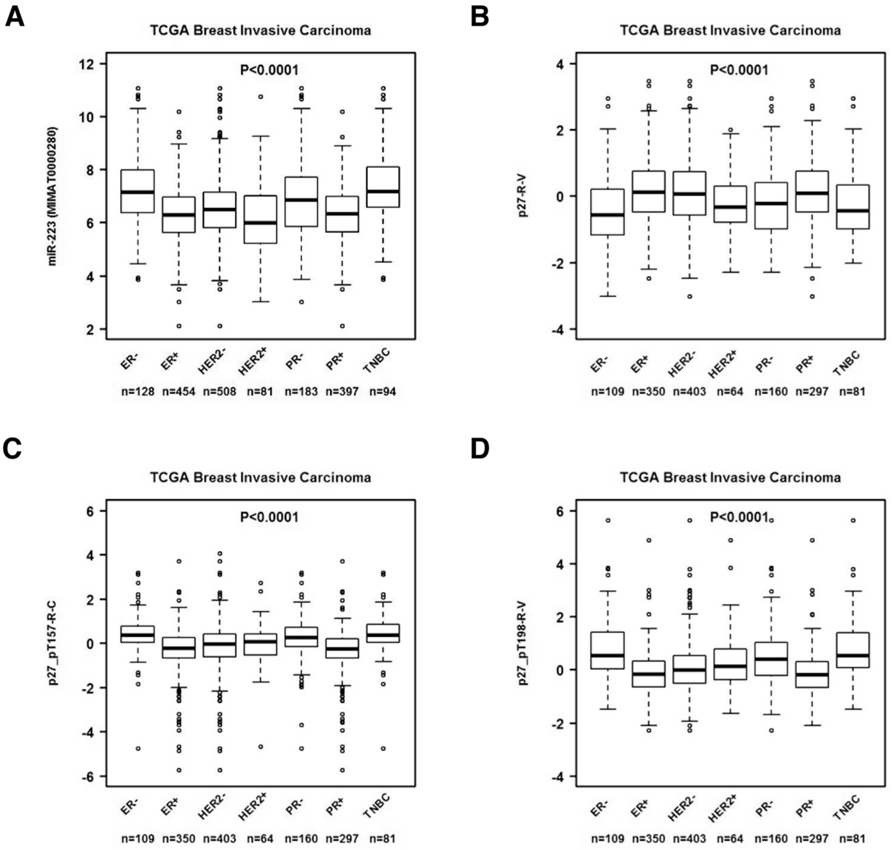

Figure S5. *Expression of miR-223 correlates with p27 T157 phosphorylation in human breast cancer.* Expression of miR-223 (**A**), p27 (**B**), p27 pT157 (**C**) and p27 pT198 (**D**) in the TGCA dataset, evaluated by microarray analyses (**A**) or RPPA (**B-D**).
