# Supplementary material for: Contact inhibition modulates intracellular levels of miR-223 in a p27kip1-dependent manner
Source: Oncotarget. 2014 Mar 4;5(5):1185–97. doi: 10.18632/oncotarget.1803 (PMC4012735; doi:10.18632/oncotarget.1803)
Supplement: Supplementary file 2 [file oncotarget-05-1185-s002.pdf]

Supplementary Data

Contact inhibition modulates intracellular levels of miR-223 in a p27kip1-dependent manner

Joshua Armenia, Linda Fabris, Francesca Lovat, Stefania Berton, Ilenia Segatto, Sara D'Andrea, Ivan Cristina, Luciano Cascione, George A. Calin, Carlo M. Croce, Alfonso Colombatti, Andrea Vecchione, Barbara Belletti and Gustavo Baldassarre.

Supplementary Materials and Methods

Cell culture

Primary wild type (WT) and p27 knock-out (p27KO) mouse embryo fibroblasts (MEFs) were prepared from embryos at day 13.5, as previously described [1].

Primary MEFs (at least 3 different preparations/genotype) were frozen at passage 1 and used in all subsequent experiments between passage 2 and 4, without significant differences. The correct genotype of WT, p27KO cells was determined by PCR, as described [1]. 3T3 fibroblasts were obtained from primary MEFs following the 3T3 immortalization protocol, as described elsewhere [2]. MEFs and 3T3 fibroblasts were cultured in Dulbecco modified Eagle medium (DMEM) supplemented with 10% fetal bovine serum (FBS) (Sigma).

Stable cell clones of 3T3 fibroblasts were obtained by retroviral transduction with a vector encoding for K-Ras4B^{V12} carrying puromycin resistance obtained by the ADDGENE consortium. The G12V oncogenic form of K-Ras4B has been used for 3T3 fibroblasts transduction. Pools were selected in complete medium supplemented with 1.5 µg/ml Puromycin. The stable expression of the different constructs was tested by Western Blot analysis of the target protein. Each experiment has been performed using at least two pools.

MDA-MB-231, HT-1080, BT-474, HBL-100, SK-BR-3, MCF-7, T47-D, MCF-10A and 293T-17 cell lines were obtained from ATCC (LGC Standards). 293FT cell line was purchased from Life Technologies (Invitrogen). Cells were all cultured in

DMEM supplemented with 10% FBS, except for MCF-10A cells, cultured in Mammary Epithelial Cell Growth Medium supplemented with bullet kit (Promo Cell).

mmu-miR-expression profile and qRT-PCR

Total RNA (5 μg) was reverse transcribed using biotin end-labeled random octamer oligonucleotide primers. Hybridization of biotin-labeled complementary DNA was performed using a custom miRNA microarray chip (OSU-CCC Human and Mouse MicroRNA Microarray Version 4.0) which contains 1,600 miRNA oligo probes derived from 474 human and 373 mouse miRNA genes and printed in duplicates. The hybridized chips were washed and processed to detect biotin-containing transcripts by streptavidin–Alexa647 conjugate, scanned and quantitated using an Axon 4000B scanner (Axon Instruments, Downingtown, PA) and the GenePix 6.0 software (Axon Instruments, Downingtown, PA). Three replicates were tested for each sample.

Average values of the replicate spots of each miRNA were background subtracted, normalized, and further analyzed. Normalization was performed using the global median method. We selected the miRNAs measured as present in at least as many samples as the smallest class in the data set (50%). Absent calls were thresholded to 6.2 (log2 scale) before statistical analysis, representing the average minimum intensity level detectable in the system. Differentially expressed miRNAs were identified using the Class Comparison Analysis of BRB tools version 3.6.0 (http://linus.nci.nih.gov/BRB-ArrayTools.html). The criterion for inclusion of a gene in the gene list is a p-value less than 0.05. The miRNA expression data have been submitted to the Gene Expression Omnibus (GEO) with accession number GSE 45538.

RNA from exponentially growing (EG), G1-blocked (G1) and S-phase (S) p27WT and p27KO MEFs used for the array data were further used for miR validation by qRT-PCR.

Vectors

Retroviral vectors (murine stem cell virus retroviral vectors, MSCV; Clontech) or EGFP-tagged vectors encoding for human $p27^{WT}$, mutant $p27^{CK-}$ (R30, L32, F62, F64 in Alanine), mutant $p27^{KR}$ (K165, R166 in Alanine), mutant $p27^{T157A}$ (T157 in Alanine), $p27^{T157D}$ (T157 in Aspartate), $p27^{Mut1}$ (R93A, P95A and K96A changing the RNA binding motif 92-PRPPKG-97 in PAPAAG), $p27^{K134fs}$ (obtained by deleting the

A420 of p27cDNA that results in the frameshift of the coding sequence of p27 starting from at K134 with a premature a stop codon at aa 143) and p27^{dMUT} (p27^{Mut1}+ p27^{K134fs}) were produced by site directed mutagenesis using a dedicate kit (Stratagene) as described elsewhere [3].

The EGFP-tagged p27 deletion mutants were previously described [1] or generated by PCR starting from human p27 cDNA using the following primers:

p27 P 1 FW: 5'-GGATCCCATATGTCAAACGTGCGAGTG-3'

p27 P 26 FW: 5'-GGATCCCATATGCCCTCGGCCTGCAGGAAC-3'

p27 P 42 FW : 5'-GGATCCCATATGACCCGGGACTTGGAGAA-3'

p27 P 78 FW : 5'-GGATCCCATATGGAGGTGGAGAAGGGCAGC-3'

p27 P 85 RV: 5'- GGATTCCTCGAGGGGCAAGCTGCCCTTCTC-3'

p27 P 154 RV: 5'-GGATCCCTCGAGTCGCTTCCTTATTCCTGC-3'

p27 P 170 RV: 5'-GGATCCCTCGAGTGTTCTGTTGGCTCTTTT-3'

p27 P 198 RV: 5'-GGATCCCTCGAGTTACGTTTGACGTCTTCT-3'

p27 K134fs FW: 5'- TTGGTGGACCCAAGACTGATCCGT-3'

p27 K134fs RV: 5'- ACGGATCAGTCTTGGGTCCACCAA-3'

The K-Ras4B^{V12} oncogene was obtained by the ADDGENE consortium and then cloned in pMSCV-hygro retroviral vector, Clontech.

Lentiviruses expressing miR-223 were produced in 293-FT cells (Invitrogen), by calcium phosphate transfection. To this purpose, the System Biosciences (SBI, USA) microRNA Precursor Constructs were used, containing expression of the specific microRNA, encoding also for the puromycin resistance for simple identification and monitoring of cells positive for transduction. In particular, MDA-MB-231 and HT-1080 cell lines were transduced with lentiviruses expressing precursor of miR223 and transduced cells were selected using 1.5 µg/ml puromycin.

Production of bacterial recombinant p27 protein was performed as previously described [1, 3]. Briefly, p27WT cDNA was cloned in the pQE-30 vector (Qiagen). Escherichia coli (M15) cells were transformed with the expression plasmid to produce recombinant proteins containing His-tag at the N-terminus. Proteins were subsequently purified with Ni-nitrilotriaceticacid (NiNTA) resin (Qiagen).

Cell Treatment and qRT-PCR

MEFs were harvested at several culture conditions: exponential growth (EG), serum starvation (ST), release in complete medium (10% FBS) for 13-15 hours (R13, R15), high confluence (HC) and high confluence followed by serum starvation (HC ST).

In the conditioned media experiment, exponentially growing p27WT MEF were grown in conditioned media harvested from exponentially growing (EG) or highly confluent (HC) MEFs. RNA was then extracted after 24 hrs for qRT-PCR analysis.

In the cell-splitting experiment, p27WT and p27KO MEFs were cultured in HC condition and then replated at high (HC, $4x10^4$ cells/cm^2 dish) or low (LC, $1x10^4$ cells/cm^2) confluence. RNA was then extracted after 4, 8 and 24 hrs for qRT-PCR analysis.

In the EGTA experiment, MEFs grown at HC were treated with 5mM EGTA. RNA from treated and untreated cells was extracted after 1 hr for qRT-PCR analysis.

In the Actinomycin D experiment, MEFs grown at HC were treated with Actinomycin D (5 mg/ml, Sigma-Aldrich). RNA was extracted at each indicated time for qRT-PCR analysis.

In the α-amanitin experiment, MEFs grown at HC were treated with α-amanitin 20µg/ml, as described in [4]. RNA was extracted at each indicated time for qRT-PCR analysis.

In the experiment of miR-223 exogenous expression, MEFs were transduced with lentiviral vectors encoding for miR-223 and 72 hrs later cells were cultured at HC and then treated with 5mM EGTA for 1 hour.

In the experiment of pri-miR223 and miR223 expression in 3T3 fibroblasts, 3T3 p27KO cells were clones were transduced with retroviral vectors encoding for p27WT, $p27CK^-$ and p27KR proteins. Exponentially growing fibroblasts were then harvested for RNA preparation and qRT-PCR analysis.

In the experiment using KRAS-transformed 3T3 fibroblast, exponentially growing 3T3-KRAS p27WT and p27KO cells were transfected with 5µg of control oligo or hsa-miR-223 using oligofectamine reagent (Invitrogen). Cell were then plated at exponential growth and high confluence and 24 hours later harvested for FACS analysis and RNA extraction.

RNA was extracted using Trizol reagent (Invitrogen) and retro-transcribed using the TaqMan MicroRNA Reverse Transcription Kit (Applied Biosystems).

MicroRNAs expression levels were quantified by qRT-PCR, using the Single Tube TaqMan microRNA Assay and the Universal Master Mix (Applied Biosystems). Normalization of the data was performed using the U6 snRNA expression. Reverse Transcriptase reactions and Real-Time PCR were performed according to the manufacturers' instructions. qRT-PCR reactions were run in the MyiQ2 Two Color Real-time PCR Detection System (Biorad). Comparative real-time PCR was performed in triplicate, including no-template controls. Relative expression was calculated using the comparative $2^{\wedge-\Delta Ct}$ method.

In the pri-miR223 experiments, RNA was retro-transcribed with AMV Reverse transcriptase to obtain cDNAs, according to provider's instruction, (Promega). Absolute quantification of pri-miR223 was evaluated by qRT-PCR using SYBR Green dye-containing reaction buffer (GoTaq qPCR 2x Master Mix, Promega). Standard curves (10-fold dilution from 10^{1} to 10^{-4} attomoles) were prepared for both pri-miR223 and housekeeping genes. The incorporation of the SYBR Green dye into the PCR products was monitored in real time using the MyiQ2 Two Color Real-time PCR Detection System (Biorad).

Ct values were converted into attomoles. Then normalized pri-miR223 value was obtained by using at least two different housekeeping genes (GAPDH, GUSB). The following primers (Sigma) were used:

mouse pri-miR223 FW: 5'-TCCAGTTGCACATCTTCCAG-3'
mouse pri-miR223 RV: 5'-TGTAGGCAGCAGGCTATGTG-3'
mouse GusB FW: 5'-CTCTGGTGGCCTTACCTGAT-3'
mouse GusB RV: 5'-CAGTTGTTGTCACCTTCACCTC-3'
mouse GAPDH FW: 5'-TGAGGACCAGGTTGTCTCCT-3'
mouse GAPDH RV 5'-CCCTGTTGCTGTAGCCGTAT-3'

Luciferase assay and mutagenesis

A 5'-flanking fragment of mouse pri-miR-223 (AK036748) according to [5] from position –758 to +23 relative to the transcription start site (+1) was amplified from mouse genomic DNA (C57/BL6 mouse), using specific primers carrying restriction endonuclease sequences XhoI (in the sense primer) and Hind III (in the antisense primer). PCR product was digested with Xho I and Hind III, unidirectionally subcloned into the promoter-less pGL3 basic vector (Promega) creating miR223 promoter full length (FL).

Putative binding sites for E2F1 transcription factor were explored by conducting the Transcription Element Search System (http://www.cbil.upenn.edu/tess/index.html) and by PROMO 3.0 (alggen.lsi.upc.es/recerca/menu_recerca.html).

For the E2F1 deleted fragments (E2F1 Del) a sense primer downstream of the two putative E2F1 binding sites with restriction endonuclease sites XhoI was used and cloned as described for FL vector.

mmu-miR-223 promoter FL F: 5'-ACGCTCGAGGGTGCTGTTACAAAGATAAGGCAAA-3'

mmu-miR-223 promoter FL R: 5'-ACGAAGCTTAAGTGGTGCCTTTGTCTTGG-3'

mmu-miR-223 promoter E2F1 Del F: 5'-ACGCTCGAGGGGAAGTCAGTGTTTTTGGAG -3'

For the point mutations of E2F1 binding sites site-directed mutagenesis of the FL vector was performed using the QuikChange Site-Directed Mutagenesis Kit (Stratagene) according to the manufacturer's instructions using the following primers:

mmu-miR-223 promoter E2F1 Mut 1 F: 5' - GCTCTGATATTTAAAGATCTCAATTGCTCTAGGG-3'

mmu-miR-223 promoter E2F1 Mut 1 R: 5' - CCCTAGAGCAATTGAGATCTTTAAATATCAGAGC-3'

mmu-miR-223 promoter E2F1 Mut 2 F: 5' - GTACTTCCTGCTTCACTCTGTAGCATG-3'

mmu-miR-223 promoter E2F1 Mut 2 R: 5' - CATGCTACAGAGTGAAGCAGGAAGTAC-3'

E2F1 Mut 3 vector was generated using E2F1 Mut 1 as template and mmu-miR-223 promoter E2F1 Mut 2 as primers for mutagenesis assay performed as described above.

For the luciferase assays, MEFs were cotransfected with 15µg of one of the reporter constructs (ppri-mir-223-full length, ppri-mir-223-E2F1-del or pGL3 basic vector) and 2.5µg of pTK-RL vector (internal control) using Lipofectamine reagent (Invitrogen) according to manufacturer's recommendations.

293-T17 cells were cotrasfected with 7µg of ppri-mir-223-full length, 7 µg of FLAG-$p27^{WT}$ or FLAG-$p27^{CK-}$ and 2µg of pTK-RL vector (internal control) using calcium phosphate method.

After transfection, cells were plated at EG and HC and 24 hrs later, cell lysates were assayed for luciferase activity using the Dual-Luciferase reporter assay system (Promega). Values were normalized using Renilla luciferase.

BrdU assay and FACS analysis

For BrdU incorporation assay, MEFs were transfected with a synthetic miR-223 inhibitor (Life Technologies) using oligofectamine reagent (Life Technologies).

Cells were then harvested at EG or at HC from 60mm dishes (BD, Falcon) containing coverslips (Menzel-Glaser, 12 mm) and were incubated for 3 hours with 10 µM BrdU (Roche).

To evaluate cell cycle entry, WT and p27KO MEF overexpressing miR-223 or control empty vector were starved and grown at high confluence (HC-ST) and then released in complete medium for 13, 16, 18 hours in the presence of 10 µM BrdU (Roche). .

Cells were then fixed in 4% paraformaldehyde (PFA) in PBS at room temperature (RT) and permeabilized in HCl 1,5 N for 30 minutes at 37°C. Coverslips were then washed 2 times in Borate Buffer 0.1 M pH 8.5 and 2 times in PBS. Incubation with primary antibody anti-BrdU (Roche) was performed 1 hour at 37°C, and samples were washed in PBS and incubated with secondary antibody (anti-mouse Alexa Fluor488-conjugated, Invitrogen) for 1 hour at RT.

Finally, nuclear staining with propidium iodide 3 µg/ml + RNase 100 µg/ml for 30 minutes at RT was performed and coverslips were mounted on glass slides with Mowiol 488 (Calbiochem).

Cell cycle distribution was analyzed by flow cytometry (FACS). WT MEF overexpressing miR-223 or control empty vector were starved and grown at high confluence (HC-ST) and then released in complete medium for 13, 16, 18 hours (R-13, R-16, R-18). 3T3-KRAS p27WT and 3T3-KRAS p27KO overexpressing miR223 or control oligo were transfected as indicated. Cells were then plated and 24 hours later harvested at exponential growth and high confluence.

Cells were then collected, fixed in ice-cold 70% ethanol, washed in PBS 1x and resuspended in propidium iodide staining solution (50 µg/ml propidium iodide and 100 µg/ml RNase A, in PBS 1X). Stained cells were subjected to FACS analysis with a FACScan or a FACSCalibur instrument (BD Biosciences). Distribution of cells in G1, S and G2/M phases of the cell cycle was calculated using the WinMDI2.8 software.

Immunofluorescence analysis

For immunofluorescence analysis EG, ST and HC MEFs and EG 3T3 p27KO fibroblasts (+p27^{WT}, +p27^{CK-}, +p27^{KR}) were harvested from 60mm dishes (BD Falcon) containing coversplips (Menzel-Glaser, 12 mm) and fixed in phosphate-buffered saline (PBS)–4% paraformaldehyde (PFA) at room temperature (RT), permeabilized in PBS–0.2% Triton X-100, and blocked in PBS–1% bovine serum albumin (BSA). Incubation with primary anti-p27 antibody (sc-528, Santa Cruz) was performed overnight at 4°C in PBS–1% BSA, then samples were washed in PBS and incubated with secondary antibodies (Alexa Fluor 488-conjugated anti-rabbit antibody, Invitrogen) for 1h at RT. Antibody incubation was followed by nuclear staining with propidium iodide solution (propidium iodide 3 µg/ml + 100 µg/ml RNase in PBS) for 30 min at RT. Stained cells were observed using a confocal laser-scanning microscope (TSP2 Leica) interfaced with a Leica DMIRE2 fluorescent microscope.

In vitro microRNA degradation assay

The *in vitro* microRNA degradation assay was essentially performed as described elsewhere [6]. Proteins from p27KO MEF cells were extracted at 4°C using degradation buffer (50mM Tris–HCl, pH 8.0, 10mM MgCl2, 75mM KCl, 5mM DTT, 10mM ascorbic acid and protease inhibitor cocktail, Roche) and after 2 cycles of freeze and thaw lysates were centrifuged to pellet debris. 5µg of RNA oligos hsa-miR223, hsa-miR1 or control miR (Sigma Aldrich) were added to 100µg of protein lysate and incubated at 25°C for 90 and 120 min with the addition of 1µg/µl recombinant p27 protein (1µg/sample) or degradation buffer as a control. At indicated times, each reaction was split in two aliquots: one aliquot for evaluation of oligo RNA levels, to which 2x loading dye (8M urea, 20mM Tris–HCl pH 8.0, 1mM EDTA, pH 8.0, 0.025% xylene cyanol FF and 0.025% bromophenol blue) was added; the other aliquot for evaluation of protein expression, to which 2x Laemli buffer was added. Samples were denatured at 95°C for 5 min. Oligo RNAs were separated on a 15% denaturing polyacrylamide gel, while total proteins were separated in 4-20% SDS-PAGE (Criterion Precast Gel, Biorad).

RNA immunoprecipitation

For prediction of RNA-binding residues of p27 protein, a search using BindN web-tool (http://bioinfo.ggc.org/bindn/) as described [7] was conducted using human and mouse p27 amino acid sequence as inputs.

Stable cell lines expressing miR223 (MDA-MB-231, HT-1080) described before, were transfected with 10µg of pEGFP-p27 expression vectors using FuGENE HD transfection reagent (Promega). 48 hours later cells were harvested for protein extraction.

RNA immunoprecipitation assay (RIP) was performed essentially as previously described [8]. To extract total proteins cells were scraped on ice using cold RIPA lysis buffer (150mM NaCl, 50mM Tris HCl pH 8.0, 1% NP40, 0.5% sodium deoxycholate, 0.1% SDS) plus a protease inhibitor cocktail (Complete™, Roche) and supplemented with 1 mM Na3VO4 (SIGMA), 1 mM DTT (SIGMA) and 100U/ml rRNasin RNase Inhibitor (Promega). The lysate was incubated on ice for 5 minutes. Protein A and protein G Sepharose 4 Fast Flow (GE Healthcare Life Sciences) beads were pre-swollen in NT2 buffer (50 mM Tris-HCl (pH 7.4), 150 mM NaCl, 1 mM MgCl2, 0.05% NP40) supplemented with 5% BSA to a final ratio of 1:5 for at least 1 h at 4 °C. Antibody anti-GFP (Roche) was added to the bead slurry and incubated overnight, tumbling end over end at 4 °C. Antibody-coated beads were washed with 1 ml of ice-cold NT2 buffer 4 times. After the final wash, beads were resuspended in 1 ml of ice-cold NT2 buffer and 200 units of rRNasin RNAse inhibitor were added. Lysates were centrifuged at 15,000g for 15 min to clear from large particles. The cleared lysate (2mg for endogenous miR223, 1mg for miR223 overexpressing cells) was added to the antibody mixture and incubated for 4 h at 4 °C tumbling end over end. Beads were then washed twice with each of the following wash buffer: low salt wash buffer (20mM Tris HCl pH 8.0, 150mM NaCl, 0.1% SDS, 1% triton X-100 and 2mM EDTA), high salt wash buffer (20mM Tris HCl pH 8.0, 500mM NaCl, 0.1% SDS, 1% triton X-100 and 2mM EDTA) and NT2 buffer supplemented with 2M Urea and 1% sodium deoxycholate. Beads were then resuspended in 1 ml of NT2 buffer and 400 µl were saved for Western blotting analysis after the addition of 3x laemmli buffer. The remaining 600 µl were used for qRT-PCR analysis: beads were pelletted down and resuspended in 50 µl of NT2 buffer supplemented with 30 µg of proteinase K. The mixture was incubated at 55°C for 30 min and Trizol reagent was then added to isolate the RNA from the immunoprecipitated pellet. The extracted RNA was

retrotranscribed using the TaqMan MicroRNA Reverse Transcription Kit and analyzed by qRT-PCR using TaqMan MicroRNA Assays, as described before. The PCR products were also separated on a 15% non denaturing-polyacrylamide gel.

Preparation of protein lysates and immunoblotting

3T3 p27KO fibroblasts overexpressing p27 (p27WT, p27CK$^-$, p27KR) and 3T3-KRAS p27WT and p27KO were grown in DMEM with 10% FBS and harvested at exponential growth.

MDA-MB-231, HT1080 cell lines overexpressing miR223 were transfected with p27 expression vectors using FuGENE HD transfection reagent (Promega), as described above and 48 hours later cells were harvested.

p27WT and p27KO MEFs were harvested at EG, HC, ST, HC+ST, ST and released in complete medium (10% FBS) for indicated time. Mammary cell lines (MDA-MB-231, BT-474, HBL-100, SK-BR-3, MCF-7, T47-D, MCF-10A) were harvested at EG or HC.

To extract total proteins, cells were scraped on ice using cold RIPA lysis buffer (150mM NaCl, 50mM Tris HCl pH 8.0, 1% NP40, 0.5% sodium deoxycholate, 0.1% SDS) or NP40 lysis buffer (0.5% NP40; 50 mM HEPES pH 7; 250 mM NaCl; 5 mM EDTA; 0.5 mM EGTA, pH 8) plus a protease inhibitor cocktail (Complete™, Roche) and supplemented with 1 mM Na3VO4 (SIGMA), 10 mM NaF (SIGMA) and 1 mM DTT (SIGMA).

For immunoblotting analysis, proteins were separated in 4-20% SDS-PAGE (Criterion Precast Gel, Biorad) and transferred to nitrocellulose membranes (GE Healthcare Life Sciences). Membranes were blocked with 5% dried milk in TBS-0.1% Tween20 or in Odyssey Blocking Buffer (Licor, Biosciences) and incubated at 4°C overnight with primary antibodies. Then, membranes were incubated 1 hour at RT with horseradish peroxidase-conjugated secondary antibody (GE Healthcare Life Sciences) for ECL detection (GE Healthcare Life Sciences) or with IR-conjugated (Alexa Fluor 680, Invitrogen or IRDye 800, Rockland) secondary antibodies for infrared detection (Odyssey Infrared Detection System, Licor). For IPs, anti-Rabbit IgG and anti-Mouse IgG True Blot (eBioscience) secondary HRP-conjugated antibodies were used.

Primary antibody were purchased from BD Biosciences: p27 (C157); from Santa Cruz: p27 (SC-C19), E2F1 (SC-193), vinculin (SC-7694), fibrillarin (SC-25397);

from R&D Systems phospho-p27(T157) (AF1555); from Roche: GFP (11814460001); from Calbiochem: Pan-Ras (OP40).

Separation of nuclear and cytoplasmic fraction

MDA-MB-231 mammary carcinoma cells overexpressing miR-223 were transfected with 10µg of pEGFP or pEGFP-p27 expression vectors using FuGENE HD transfection reagent (Promega). 48 hours later cells were harvested for protein extraction. To perform the differential extraction of cytoplasmic and nuclear proteins, cells were resuspended in Buffer A (10 mM HEPES pH 7.9, 0.1 mM EDTA pH 8, 0.1 mM EGTA pH 8, 10 mM KCl) plus all the previously described inhibitors. Samples were kept on ice for 15 minutes, after that 0.5% NP40 was added and samples were centrifuged at 6000 rpm for 1 minute at 4°C. The supernatant, representing the cytoplasmic protein fraction, was collected.

After three washes in Wash Buffer (0.32M sucrose, 3mM CaCl2, 2mM Mg-Acetate, 0.1mM EDTA, 10mM Tris HCl pH8, 0.5% NP40), pellets were digested in Buffer C (20 mM HEPES pH 7.9, 1 mM EDTA pH 8, 1 mM EGTA pH 8, 400 mM NaCl, plus all the inhibitors) and incubated on ice for 20 minutes. Samples were then centrifuged at max speed for 15 minutes at 4°C to recover the supernatant, representing the nuclear protein fraction. Cytosolic and nuclear fractions were stored for subsequent RIP assay as described above.

TCGA statistical analyses

We downloaded clinical, miRNA expression and Reverse Phase Protein Array (RPPA) data from TCGA (The Cancer Genome Atlas) available through the associated files of the paper Comprehensive molecular portraits of human breast tumors, Nature, September 27, 2012 https://tcga-data.nci.nih.gov/docs/publications/brca_2012/

Statistical analyses were performed in R (version 2.14.2) (http:///www.r-project.org/). All tests were two-sided and considered statistical significant at the 0.05 level. The Log-rank test was employed to determine the significance of the association between miR-223 (MIMAT0000280) expression and total p27, p27 T157 and p27 T198 RPPA. The Spearman's rank-order correlation test was applied to measure the strength of the association between miR-223 expression and p27T157 RPPA levels.

The Shapiro-Wilk test was applied to determine whether data followed a normal distribution. The analysis of variance test was applied to normally distributed data, otherwise the Kruskal-Wallis test was applied to assess the association of miRNA/RPPA levels with breast tumor subtypes.

Supplementary References

1. Baldassarre G, Belletti B, Nicoloso MS, Schiappacassi M, Vecchione A, Spessotto P, Morrione A, Canzonieri V, Colombatti A. p27(Kip1)-stathmin interaction influences sarcoma cell migration and invasion. Cancer Cell. 2005;7:51-63
2. Todaro GJ, Green H. Quantitative studies of the growth of mouse embryo cells in culture and their development into established lines. J. Cell Biol. 1963;17: 299-313.
3. Schiappacassi M, Lovisa S, Lovat F, Fabris L, Colombatti A, Belletti B, Baldassarre G. Role of T198 modification in the regulation of p27(Kip1) protein stability and function. PLoS One. 2011, e17673.
4. Chen AJ, Paik JH, Zhang H, Shukla SA, Mortensen R, Hu J, Ying H, Hu B, Hurt J, Farny N, Dong C, Xiao Y, Wang YA, Silver PA, Chin L, Vasudevan S, Depinho RA. STAR RNA-binding protein Quaking suppresses cancer via stabilization of specific miRNA. Genes Dev. 2012; 26: 1459-72.
5. Fukao T, Fukuda Y, Kiga K, Sharif J, Hino K, Enomoto Y, Kawamura A, Nakamura K, Takeuchi T, Tanabe M. An evolutionarily conserved mechanism for microRNA-223 expression revealed by microRNA gene profiling. Cell. 2007;129:617-31.
6. Lu S, Sun YH, Chiang VL. Adenylation of plant miRNAs. Nucleic Acids Res. 2009;37, 1878-85.
7. Wang L, Brown SJ. BindN: a web-based tool for efficient prediction of DNA and RNA binding sites in amino acid sequences. Nucleic Acids Res. 2006;34: 243-48.
8. Keene JD, Komisarow JM, Friedersdorf MB. RIP-Chip: the isolation and identification of mRNAs, microRNAs and protein components of ribonucleoprotein complexes from cell extracts. Nat Protoc. 2006;1:302-07.
